# Health technology assessment in Eastern Europe and Central Asia: an updated SWOT analysis

**DOI:** 10.1017/S0266462326103614

**Published:** 2026-03-30

**Authors:** Rabia Sucu, Antonio Migliore, Nicola Vicari, Esra Meltem Koç, Alima Almadiyeva

**Affiliations:** 1 Health Technology Assessment International, Canada; 2 İzmir Katip Çelebi Üniversitesi İslami İlimler Fakültesi, Türkiye

**Keywords:** Health technology assessment, EECA, regional collaboration, evidence-based decision making, SWOT

## Abstract

Health technology assessment (HTA) is increasingly recognized as a critical tool for evidence-informed decision-making in Eastern Europe and Central Asia (EECA), a region characterized by substantial diversity in health system maturity, financial resources, and institutional capacities. Building on the previous Eurasian HTA Initiative conducted a decade earlier, this study aimed to update the regional assessment of HTA implementation, expand country representation, and identify evolving strengths, weaknesses, opportunities, and threats affecting the advancement of HTA across the region. An online multilingual survey, informed by the original SWOT framework, was disseminated between August and October 2025 to experts from national authorities, HTA agencies, academia, and health sector stakeholders across eleven EECA countries. Twenty-two responses were analyzed descriptively. Key strengths included strong regional collaboration interest, growing availability of online HTA resources, and sustained global support. Major weaknesses centered on the limited trained HTA/EBM personnel and the absence of standardized national training programs. Opportunities reflected expanding interest in EBM, pressure from rising healthcare costs, and prospects for regional advocacy, educational exchanges, and international collaboration. Principal threats involved insufficient funding for capacity building, low policy-maker and clinician awareness, commercial influence from industry, and limited incentives for EBM uptake. Overall, respondents emphasized gradual progress in selected areas but persistent structural barriers requiring coordinated national and regional action.

## Introduction

Eastern Europe and Central Asia (EECA) encompasses a broad set of countries with diverse historical, political, and economic backgrounds that shape their healthcare systems. This diversity includes European Union (EU) Member States, such as Poland, Greece, and Slovenia, alongside upper-middle-income countries like Kazakhstan, Georgia, Serbia, and Ukraine, as well as lower-middle-income nations including Kyrgyzstan, Uzbekistan, and Tajikistan (1). Many states in the region have faced persistent challenges, particularly chronic underfunding of healthcare services. As a result, patients are often burdened with substantial out-of-pocket payments, a situation worsened by limited public health insurance coverage and inefficient allocation of healthcare resources (2). Like many other countries in the world, EECA countries are confronted with high levels of noncommunicable diseases (3) occurring simultaneously with ongoing issues related to infectious diseases (4). In addition to that, shortages of healthcare professionals, outdated infrastructure, and political and economic instability further undermine long-term system planning and reform efforts (5). Nevertheless, awareness of the value of health technology assessment (HTA) is growing throughout EECA (6;7). As a vital instrument for evidence-based decision-making, especially in resource-constrained contexts, HTA supports more efficient allocation of resources (8) by offering a structured method to evaluate healthcare interventions and guide policymakers toward those that deliver the greatest benefits relative to their costs (9). Approximately ten years ago, the Eurasian HTA Initiative was established under the leadership of the Turkish Evidence Based Medicine Society, involving nine EECA countries to analyze common strengths, weaknesses, opportunities, and threats (SWOT) related to HTA implementation and evidence-informed decision-making. A SWOT analysis was chosen as a structured and participatory approach to assess the state of HTA implementation, governance, capacity, and integration within healthcare decision-making processes (10;11). By engaging diverse stakeholders, including policymakers, HTA specialists, and healthcare professionals, this method helped uncover key enablers and barriers, supporting a comprehensive understanding of both country-specific and regional HTA landscapes (12). The project’s outcome was presented across several events, but no results were ever published in scientific literature. Building on this foundation, a project was initiated by HTAi with the following objectives: (i) To update the assessment of HTA and evidence-informed decision-making implementation in EECA countries, reflecting developments since the Eurasian HTA Initiative; (ii) To expand regional representation by including additional countries; (iii) To identify ongoing challenges and emerging opportunities for HTA capacity building, policy integration, and regional collaboration; (iv) To provide actionable recommendations for advancing HTA practices and evidence-based health policies in the region.

## Methods

A structured, cross-sectional expert survey based on a predefined SWOT framework was used to map perceptions of HTA and EBM development across EECA countries. Microsoft Forms was used to implement the questionnaire. Responses were collected from 11 August 2025 to 16 October 2025. Multiple reminders were sent to encourage participation. The questionnaire was deliberately built on the earlier Eurasian HTA Initiative SWOT list and administered online in three languages (English, Kazakh, and Russian) to HTA, EBM, and policy stakeholders identified through national networks and HTAi activities (13;14) (convenience sampling). The primary aim was to capture informed practice-oriented views from those directly involved in HTA-related processes, rather than to obtain a statistically representative sample of the whole stakeholders’ ecosystem. Each SWOT element was rated on a four-point Likert scale for current relevance and on a three-category scale for perceived change over time, which allowed translation of expert judgements into simple quantitative indicators while preserving the ordinal nature of responses. The choice of descriptive statistics reflects both the small sample size and the exploratory purpose of the work: the goal was to identify dominant patterns and priority issues, not to test hypotheses or estimate precise effect sizes. The open-ended comment fields were incorporated to apprehend contextual details that could not be adequately reflected in numeric ratings. Overall, the choice of methodological tools was made to balance the feasibility in a resource-constrained, multi-country context and the need to generate a systematic, comparable, and policy-relevant picture of the strengths, weaknesses, opportunities, and threats surrounding HTA and EBM in the region.

## Results

### Respondents’ analytics (details are presented as Supplementary Material)

#### Representativeness

A total of twenty two responses were collected from eleven countries: Albania, Hungary, Kazakhstan, Kyrgyzstan, Moldova, Montenegro, North Macedonia, Poland, Serbia, Türkiye, Ukraine. The data set included, in some cases, multiple respondents from each country (for example, Kazakhstan, Serbia, and Türkiye). The respondents represent a diverse range of organizational and professional perspectives relevant to HTA implementation, including academic institutions, government entities, public institutions, such as national agencies or research centers, consultancy firms, nongovernmental organizations, hospitals, pharmaceutical companies, and other private entities. Most respondents reported substantial familiarity with HTA implementation in their countries. Specifically, 68.2 percent (n = 15) and 22.7 percent (n = 5) indicated they were “very familiar” or “somehow familiar” with HTA implementation. Only two respondents (9 percent) reported slight or no familiarity with HTA implementation.

#### Formal HTA institutionalization

Although only 18.2 percent (n = 4) of respondents reported an absence of any formally appointed HTA entity in their jurisdiction, nearly one-third of respondents (31.8 percent, n = 7) indicated the situation was “complicated,” reflecting partial, transitional, or ambiguous institutional arrangements. In particular, respondents from Moldova noted that although the National Health Insurance Company had been nominated by government’s decision to assume responsibility for HTA processes and implementation, formal approval of the national HTA mechanism was pending. In Serbia, an HTA department at the Institute of Public Health addresses medical devices and procedures under a legal mandate, while responsibility for medicines resides with the Sector for Pharmacoeconomics at the National Health Insurance Fund, although the latter work is not formally recognized as HTA. In Kyrgyzstan, HTA activities are conducted through an interdisciplinary team applying evidence-based assessments, yet without formal institutional designation. For two countries, Türkiye and Kazakhstan, respondents presented conflicting opinions. Three out of the five respondents from Türkiye described the situation as “complicated” and provided detailed explanations reflecting overlapping roles, institutional ambiguity, and transitional arrangements within the Ministry of Health and related agencies. Although the Department of Health Technology Assessment operates under the Ministry of Health and can be named as a leading structure, respondents clarified that it is not fully independent. Additional narrative from respondents points to procedural complexity and the coexistence of multiple HTA-related bodies, without clear legal mandates or operational guidelines. Most respondents from Kazakhstan, six out of seven, selected “Yes” and specified the name of the main HTA institution, while one respondent described the situation as “complicated” and was not familiar with the process of HTA implementation.

#### Respondents’ involvement in HTA implementation

Respondents described varied levels and types of involvement in HTA implementation in their countries. Several respondents held or had held strategic roles, including government positions (e.g., Deputy Minister of Health, government advisors), and leadership positions within HTA departments. Academic respondents indicated roles in teaching HTA principles and participating in EBM teams. Consultancy professionals described organizing annual multistakeholder convenings to foster knowledge exchange. Only a minority of respondents reported no current involvement.

### Relevance of the SWOT elements

Elements for which at least 70 percent of respondents indicated “relevant” or “very relevant” are presented below for the four dimensions and included in Table 1. All elements are presented in detail as Supplementary Material.

**Table 1.** SWOT elements for which more than 70 percent of respondents indicated “Relevant” or “Very Relevant”

	**Relevance^a^(%)**		**Relevance** ^a^**(%)**
**STRENGTHS**		**WEAKNESSES**	
S1. Strong interest in regional collaboration, including cross-country HTA activities	**73**	W1. Shortage of trained professionals in evidence-based medicine and HTA	**82**
S3. Availability of online resources and educational materials	**73**	W2. Lack of standardized, certified HTA/evidence-based medicine programs across country	**73**
S7. Global support and partnerships (e.g. World Health Organization, World Bank) backing HTA expansion	**73**		
**OPPORTUNITIES**		**THREATS**	
O1. Growing national and professional interest in evidence-based medicine	**86**	T1. Insufficient funding for evidence-based medicine/HTA capacity development	**82**
O2. Rising healthcare costs, prompting demand for resource optimization	**91**	T2. Low awareness or resistance among policy-makers and clinicians	**77**
O4. Potential for joint policy advocacy and regional position statements on HTA	**73**	T3. Strong influence of the pharmaceutical and medical device industries	**82**
O5. Educational exchange opportunities and training via webinars, workshops, etc.	**82**	T4. Lack of incentives for physicians to adopt evidence-based medicine practices	**77**
O7. International collaboration for sharing experience and technical support	**77**		

a “Relevant” or “Very Relevant.”

#### Strengths

##### S1. Strong interest in regional collaboration, including cross-country HTA activities

This element was assessed as relevant by a substantial majority of respondents, with 73 percent rating it as “very relevant” or “relevant.” More than half (55 percent) indicated improvement in regional HTA collaboration over the past decade. Qualitative comments emphasized that much of this progress is informal, with activities strongly influenced by individual actors rather than institutionalized mechanisms. Respondents noted the potential for further development if structured collaborations and regional networks are reinforced.

##### S3. Availability of online resources and educational materials

This strength was rated highly, with 73 percent of respondents finding online HTA resources and educational materials “very relevant” or “relevant” to national HTA progress. Notably, 59 percent perceived improvements in their availability over recent years. However, multiple comments indicated that online resources, while increasingly accessible, have yet to be systematically integrated into professional development programs or national strategies.

##### S7. Global support and partnerships (e.g. World Health Organization, World Bank) backing HTA expansion

International partnerships and donor support were rated as “very relevant” or “relevant” by 73 percent of respondents. However, 68 percent reported little to no recent change in the nature or impact of global support, with some noting that external contributions were most critical during earlier stages of HTA development. As national capacity expands, future international collaboration may play a more strategic and less operational role.

#### Weaknesses

##### W1. Shortage of trained professionals in EBM and HTA

This weakness was rated as “relevant” or “very relevant” by 82 percent of respondents, highlighting workforce limitations as a principal challenge. Most (73 percent) reported no substantial change over time, although only 14 percent noted improvement. Comments revealed that although EBM is increasingly recognized and promoted, especially in Türkiye, comprehensive national capacity in both HTA and EBM remains limited. Several respondents emphasized the urgent need for expert development in HTA.

##### W2. Lack of standardized, certified HTA/EBM programs across country

A similar majority (73 percent) considered this issue “relevant” or “very relevant”; most (68 percent) saw little or no change, and only 23 percent noted improvement. Respondents described a persistent lack of formal certification schemes, with improvements stemming mainly from increased EBM exposure in undergraduate medical curricula. The absence of standardized, national programs for HTA education remains a significant barrier, with calls for novel and enhanced tools at both university and country levels.

#### Opportunities

##### O1. Growing national and professional interest in evidence-based medicine

This was strongly supported, with 86 percent of respondents rating this opportunity as “relevant” or “very relevant.” The majority (77 percent) also perceived improvement in recent years. Comments reflect increased attention to EBM, especially in academic settings and clinical practice, but note that institutionalization of EBM within national HTA frameworks is still evolving.

##### O2. Rising healthcare costs, prompting demand for resource optimization

There was marked consensus on the relevance of this pressure: 91 percent deemed rising healthcare costs “relevant” or “very relevant” as a driver for HTA adoption. Some respondents (45 percent) reported improved opportunities, although others saw either stagnation or increased challenges. Comments from Türkiye highlight how increased financial pressures in the health system are making HTA a critical tool for sustaining service delivery.

##### O4. Potential for joint policy advocacy and regional position statements on HTA

Most respondents (73 percent) saw advocacy and position statements as “relevant” or “very relevant,” but 68 percent reported stability with little improvement. Several respondents highlighted Türkiye’s potential as a regional leader but noted this has yet to be effectively harnessed for joint advocacy or policymaking.

##### O5. Educational exchange opportunities and training via webinars, workshops, etc

A high proportion (82 percent) considered educational exchanges “relevant” or “very relevant,” with 59 percent indicating improved opportunity. Comments emphasized that although webinars and workshops are widely available, targeted institutional uptake and better organization are needed to maximize capacity-building and impact.

##### O7. International collaboration for sharing experience and technical support

Most respondents (77 percent) judged international collaboration to be a “relevant” or “very relevant” opportunity. Positive change was reported by half, although the remainder saw little movement. Comments pointed to project-based and ad-hoc collaborations (especially at the EU level) as examples but stressed the need for more systematic and sustained multinational partnerships.

#### Threats

##### T1. Insufficient funding for EBM/HTA capacity development

This threat was strongly endorsed, with 82 percent of respondents considering insufficient funding “relevant” or “very relevant.” Most (50 percent) reported stagnation, 32 percent noted worsening, and only 18 percent saw improvement. Comments highlighted the chronic reliance on short-term or externally funded projects rather than sustainable national financing for HTA/EBM capacity.

##### T2. Low awareness or resistance among policy-makers and clinicians

Most respondents (77 percent) viewed this threat as “relevant” or “very relevant,” with half reporting unchanged conditions and a minority either worsening or improving. Comments noted persistent low awareness among policy-makers and minimal integration into daily clinical practice, describing interaction levels between HTA experts and decision-makers as suboptimal.

##### T3. Strong influence of the pharmaceutical and medical device industries

The majority agreement (82 percent) placed industry influence as a principal threat. Most observed little change, but 32 percent saw improvement and 18 percent noted further worsening. Comments highlighted gaps in HTA structures and oversight, allowing nonsystematic evidence to be outweighed by commercial interests in market access and reimbursement decision-making.

##### T4. Lack of incentives for physicians to adopt EBM practices

Respondents broadly recognized lack of incentives as a threat (77 percent), with half reporting stagnation, 32 percent improvement, and 14 percent worsening. Comments indicated that most physicians in Türkiye apply evidence informally and that there are no requirements or formal incentives in place for regular EBM adoption.

## Limitations

The present study has several limitations that should be acknowledged when interpreting the findings. First, the study reflects perceived strengths, weaknesses, opportunities and threats rather than objective performance indicators of HTA systems and, therefore, should be interpreted as an exploratory snapshot of views of a small group of engaged experts in EECA, not as a comprehensive or statistically generalizable assessment of the included region. Although survey participants ranged across the region, some countries were not represented, or invited representatives did not contribute to the study, despite multiple reminders (Armenia, Azerbaijan, Bosnia-Herzegovina, Georgia, Greece, Romania, Slovakia, Slovenia, and Uzbekistan). Variation in awareness, comprehension, and maturity of HTA at the national level or the unavailability of the invited experts may be the reason behind this issue. The survey relied on a small convenience sample of HTA and EBM experts taken largely from existing formal and informal networks, which introduces selection bias toward individuals who are already engaged and generally favorable to HTA. The analysis was deliberately descriptive because differences in perceptions across countries, HTA maturity levels, or stakeholder types were not explored. Qualitative comments given by experts were used illustratively without a formal thematic analysis, so the depth of interpretation is limited. Nonetheless, the elements and insights generated through the present project offer a robust qualitative foundation for regional HTA strategy development.

## Discussion

The findings of this updated regional assessment reinforce that HTA in EECA continues to evolve but remains unevenly institutionalized across countries. Progress in several areas, such as the increasing availability of educational materials and greater professional interest in evidence-informed decision-making, reflects global trends in emerging HTA systems, where early advances are often driven by individual champions rather than embedded institutional mandates. This pattern has been observed in LMIC contexts, where HTA is frequently introduced through external initiatives but struggles to achieve long-term integration without stable governance structures (15). The EECA region’s experience therefore mirrors broader international evidence showing that sustainable HTA implementation requires formalization, predictable funding, and cross-sectoral ownership rather than ad-hoc or personality-driven arrangements. A persistent challenge concerns the shortage of trained HTA professionals and the absence of standardized education pathways. Workforce limitations have been repeatedly identified as bottlenecks in LMIC and transition economies, where few universities offer structured HTA programs and most expertise is concentrated among individuals with international training. Similar to findings in sub-Saharan Africa and parts of Asia, EECA countries face gaps across the entire educational continuum, from undergraduate exposure to EBM to specialized postgraduate training (16). Without systematic investment in human resources and academic infrastructure, national HTA systems cannot achieve the critical mass needed for routine policy integration. Weak legal and governance structures further constrain the integration of HTA into routine policymaking. Many EECA countries lack clear mandates for HTA or defined processes for linking evidence to reimbursement and benefit package decisions. This finding is consistent with global mapping studies showing that policy frameworks, rather than technical capacity alone, are the strongest predictors of functional HTA systems (17). International consensus documents emphasize that HTA requires institutionalized procedures, transparency mechanisms, and stakeholder involvement to influence policy effectively (18). The EECA region’s fragmented governance arrangements, therefore, represent a major barrier to achieving the full benefits of HTA. Opportunities highlighted by respondents, including rising healthcare costs, growing demand for resource optimization, and increased attention to EBM, are well aligned with global pressures on health systems. Countries facing fiscal constraints often adopt HTA as a strategy to improve value for money, particularly where out-of-pocket expenditures remain high (19). Since international organizations such as the WHO and the World Bank continue to promote HTA as a core component of Universal Health Coverage reforms, a trend observed in many middle-income regions (20), such global momentum offers EECA countries an opportunity to anchor domestic reforms within a broader international framework. However, numerous threats risk hindering HTA progress. Respondents emphasized insufficient funding, low awareness among policymakers, and substantial influence of commercial actors, findings consistent with analyses from multiple middle-income regions where the private sector can shape market access in the absence of strong HTA governance (21). Cultural barriers and hierarchical professional norms also mirror broader post-Soviet health system dynamics, where reforms often struggle against entrenched practices (5). Discontinuity of projects and frequent staff turnover, problems widely documented in capacity-building initiatives, pose additional risks to the sustainability of HTA institutions. Addressing these structural challenges requires long-term political commitment, stable financing, and deliberate strategies to embed HTA within national policy processes.

## Key recommendations

To strengthen HTA implementation and evidence-informed decision-making across EECA, countries should prioritize establishing formal governance structures and clear legal mandates for HTA, invest in sustained human resource development through standardized training and integration of EBM in medical education, and secure predictable financing for institutional and capacity-building activities. Strengthening regional collaboration (e.g., through joint assessments, shared methodological resources, and coordinated policy dialogue) will help harmonize practices and reduce duplication of efforts. Equally important is improving access to high-quality HTA tools and guidance, including translation of key materials into regional languages, and systematically engaging clinicians, patient groups, and professional societies to foster acceptance and shared ownership of HTA processes. Together, these actions can create resilient HTA ecosystems capable of supporting transparent, equitable, and sustainable health policy decisions across the region. In this context, international organizations such as HTAi can play a critical enabling role by providing neutral platforms for cross-country dialogue, offering access to global expertise, and supporting the development of regionally adapted training and methodological resources. HTAi’s convening power, through scientific meetings, policy forums (22), and regional initiatives (14), facilitates knowledge exchange, fosters professional communities, and helps align national efforts with international best practices. Moreover, HTAi can support capacity building by connecting emerging HTA programs with established agencies, promoting mentorship and collaborative learning, and assisting countries in articulating shared regional priorities. By serving as both a knowledge broker and a catalyst for collaboration (23), HTAi can help accelerate the institutionalization and maturity of HTA systems across EECA.

## Supporting information

10.1017/S0266462326103614.sm001 Sucu et al. supplementary material Sucu et al. supplementary material

## Supplementary material

The supplementary material for this article can be found at http://doi.org/10.1017/S0266462326103614.
