# Health technology assessment in Eastern Europe and Central Asia: an updated SWOT analysis – CORRIGENDUM

**DOI:** 10.1017/S0266462326103973

**Published:** 2026-08-10

**Authors:** Rabia Sucu, Antonio Migliore, Nicola Vicari, Esra Meltem Koç, Alima Almadiyeva

**Affiliations:** 1 Health Technology Assessment International, Canada; 2 Faculty of Medicine, Department of Family Medicine, İzmir Katip Çelebi University, Türkiye

Professor Esra Meltem Koç was originally published with the affiliation:

İzmir Katip Çelebi Üniversitesi İslami İlimler Fakültesi, Türkiye

The authors correct affiliation is:

Faculty of Medicine, Department of Family Medicine, İzmir Katip Çelebi University, Türkiye

This has now been corrected on the original manuscript.

The authors apologise for this error.
