# Supplementary material for: Health technology assessment in Eastern Europe and Central Asia: an updated SWOT analysis
Source: Int J Technol Assess Health Care. 2026 Mar 30;42(1):e31. doi: 10.1017/S0266462326103614 (PMC13071842; doi:10.1017/S0266462326103614)
Supplement: Sucu et al. supplementary material [file S0266462326103614sup001.docx]

## Supplementary material

### Respondents’ analytics

A total of 22 responses were collected from 11 countries: Albania, Hungary, Kazakhstan, Kyrgyzstan, Moldova, Montenegro, North Macedonia, Poland, Serbia, Türkiye, Ukraine. The dataset included, in some cases, multiple respondents from each country (for example, Kazakhstan, Serbia, and Türkiye). The respondents represent a diverse range of organizational and professional perspectives relevant to HTA implementation with 3 participants (13.6%) from academic institutions, 5 (22.7%) from government entities (i.e., Ministries of Health), and 7 (31.8%) from public institutions, such as national agencies or research centres. The remaining 7 respondents (31.8%) were classified as “Other,” representing consultancy companies, NGOs, private organizations, hospitals, pharmaceutical companies, and private research institutes. In terms of stakeholder categories, the largest groups were Academic/Researcher (5 respondents, 22.7%) and Government Officials (5 respondents, 22.7%). HTA Agency Representatives constituted 3 respondents (13.6%), and both Consultancy and Decision Maker categories included 2 respondents each (9.1%). The remaining stakeholder groups, Clinician/Health Care Provider, Hospital-based HTA, Payer, and Pharmaceutical Company, each accounted for 1 respondent (4.5% each). The “Other” category (2 respondents, 9.1%) was comprised of an Academic / Researcher & Consultancy professional and a representative from a National Health Insurance Company. Most respondents reported substantial familiarity with HTA implementation in their countries. Specifically, 68.2% (n = 15) and 22.7% (n = 5) indicated they were “very familiar” or “somehow familiar” with HTA implementation. Only 2 respondents (9%) reported slight or no familiarity with HTA implementation.

### Formal HTA institutionalization

While only 18.2% (n = 4) of respondents reported an absence of any formally appointed HTA entity in their jurisdiction, nearly one-third of respondents (31.8%, n = 7) indicated the situation was “complicated”, reflecting partial, transitional, or ambiguous institutional arrangements. In particular, respondents from Moldova noted that although the National Health Insurance Company had been nominated by government’s decision to assume responsibility for HTA processes and implementation, formal approval of the national HTA mechanism was pending. In Serbia, an HTA department at the Institute of Public Health addresses medical devices and procedures under a legal mandate, while responsibility for medicines resides with the Sector for Pharmacoeconomics at the National Health Insurance Fund, although the latter work is not formally recognized as HTA. In Kyrgyzstan, HTA activities are conducted through an interdisciplinary team applying evidence-based assessments, yet without formal institutional designation. For two countries, Türkiye and Kazakhstan, respondents presented conflicting opinions. Three out of the five respondents from Türkiye described the situation as “complicated” and provided detailed explanations reflecting overlapping roles, institutional ambiguity, and transitional arrangements within the Ministry of Health and related agencies. Although the Department of Health Technology Assessment operates under the Ministry of Health and can be named as a leading structure, respondents clarified that it is not fully independent. Additional narrative from respondents point to procedural complexity and the coexistence of multiple HTA-related bodies, without clear legal mandates or operational guidelines. Most respondents from Kazakhstan, six out of seven, selected “Yes” and specified the name of the main HTA institution, while one respondent described the situation as “complicated” and was not familiar with the process of HTA implementation. Respondents consistently indicated that this center is formally appointed and recognized as the main expert body for HTA in Kazakhstan, with responsibilities defined by national rules and regulations.

### Respondents’ involvement in HTA implementation

Respondents described varied levels and types of involvement in HTA implementation in their countries. Those directly engaged in HTA operations described roles ranging from operational management and ecosystem development at the national level (Ukraine) to hospital-based HTA and pharmacoeconomic analysis (Kazakhstan). Several respondents held or had held strategic roles, including government positions (e.g., Deputy Minister of Health, government advisors), and leadership positions within HTA departments (Serbia, Kazakhstan). Academic respondents indicated roles in teaching HTA principles and participating in EBM teams. Consultancy professionals described organizing annual multistakeholder convenings to foster knowledge exchange. One respondent from a research institute reported prior employment at an HTA office followed by transition to independent research institute work. Only a minority of respondents reported no current involvement (North Macedonia and Kazakhstan).

### SWOT dimensions (all elements)

#### Strengths (Table S1)

*S1. Strong interest in regional collaboration, including cross-country HTA activities*

This element was assessed as relevant by a substantial majority of respondents, with 72.7% rating it as “very relevant” or “relevant.” More than half (54.5%) indicated improvement in regional HTA collaboration over the past decade. Qualitative comments emphasized that much of this progress is informal, with activities strongly influenced by individual actors rather than institutionalized mechanisms. Respondents noted the potential for further development if structured collaborations and regional networks are reinforced.

*S2. Political and institutional will through World Health Assembly (WHA) resolution promoting HTA*

The perceived relevance of the WHA resolution and broader institutional will was more mixed, with only 41% considering it “very relevant” or “relevant.” Half of respondents saw little change or a decline in the impact of these global drivers. Several participants highlighted that, although the WHA resolution exists, aligning national policies with its goals remains challenging. International support is important, but stronger domestic ownership and adaptation are needed for HTA momentum.

*S3. Availability of online resources and educational materials*

This strength was rated highly, with 73% of respondents finding online HTA resources and educational materials “very relevant” or “relevant” to national HTA progress. Notably, 59% perceived improvements in their availability over recent years. However, multiple comments indicated that online resources, while increasingly accessible, have yet to be systematically integrated into professional development programs or national strategies.

*S4. Existing training programs (e.g. pharmacoinformatics, critical appraisal) for health professionals in some countries*

Existing training programs for HTA-related skills were considered “very relevant” or “relevant” by 68% of respondents, but most (54%) reported limited or stagnant progress. Respondents described the sporadic nature of these programs, which are often embedded in academic settings and not broadly accessible as standalone modules for HTA capacity building. Ongoing investment in structured, national-level training is recognized as essential.

*S5. Capacity-building efforts supported by both national and regional actors*

Responses highlighted persistent variability: only a third of participants judged capacity-building efforts as “very relevant.” Nearly 59% reported no substantive change over time, pointing to frequent reliance on ad-hoc initiatives and external projects rather than sustained, strategic actions at the national level.

*S6. Presence of Evidence-Based Medicine (EBM) associations in the country offering peer motivation*

The emergence of EBM associations and their contribution to HTA was recognized as a strength by 68% of respondents. Comments revealed an appreciation for their role in fostering peer motivation, while also noting that their direct influence over policy is still constrained in several countries. Further institutional linkages are needed to translate professional engagement into national-level HTA advancement.

*S7. Global support and partnerships (e.g. World Health Organization, World Bank) backing HTA expansion*

International partnerships and donor support were rated as “very relevant” or “relevant” by 73% of respondents. However, 68% reported little to no recent change in the nature or impact of global support, with some noting that external contributions were most critical during earlier stages of HTA development. As national capacity expands, future international collaboration may play a more strategic and less operational role.

#### Weaknesses (Table S2)

*W1. Shortage of trained professionals in EBM and HTA*

This weakness was rated as “relevant” or “very relevant” by 82% of respondents, highlighting workforce limitations as a principal challenge. Most (73%) reported no substantial change over time, while only 14% noted improvement. Comments revealed that although EBM is increasingly recognized and promoted, especially in Türkiye, comprehensive national capacity in both HTA and EBM remains limited. Several respondents emphasized the urgent need for expert development in HTA.

*W2. Lack of standardized, certified HTA/EBM programs across country*

A similar majority (73%) considered this issue “relevant” or “very relevant”; most (68%) saw little or no change, and only 23% noted improvement. Respondents described a persistent lack of formal certification schemes, with improvements stemming mainly from increased EBM exposure in undergraduate medical curricula. The absence of standardized, national programs for HTA education remains a significant barrier, with calls for novel and enhanced tools at both university and country levels.

*W3. Absence of EBM in academic curricula at all educational levels*

While 59% found this weakness to be “relevant” or “very relevant,” 19% considered it “not at all” relevant, reflecting heterogeneity in educational practices. Perceived improvement (50%) slightly outweighed stagnation, and qualitative comments noted increases in undergraduate EBM offerings. However, concerns were raised about the quality of programs and the lack of qualified instructors, as well as EBM’s continued absence at postgraduate and continuing education levels.

*W4. Over-reliance on external expertise due to lack of national capacity*

Opinions on this statement were mixed, with only half citing “relevant” or “very relevant,” a significant 36% “not at all” relevant, and the remainder “somehow relevant.” While 59% reported no change, 27% saw improvement. Some participants described shifting away from external reliance as national capacity slowly develops, though others reported that limited policy use of HTA currently reduces both internal and external demand. The need for continued internal capacity-building was stressed.

*W5. Language barriers limiting accessibility to core documents and guidelines*

The relevance of language limitations was more variable; 64% considered them “relevant” or “very relevant,” while 27% said “not at all” relevant. Nearly half (55%) observed stagnation, 41% perceived improvement, and only 5% saw worsening. Respondents noted the absence of core HTA resources in national languages, particularly Russian and Turkish, which restricts accessibility for many professionals and decision-makers.

*W6. Missing or weak legal frameworks for institutionalizing HTA*

Most respondents (64%) found this a “relevant” or “very relevant” weakness, with 64% reporting no change and 27% citing modest improvement. The absence or ambiguity of institutional mandates for HTA in legal and regulatory structures was frequently cited as a barrier to systematic implementation. Several comments stressed the need for further policy development and strengthening of regulatory instruments.

*W7. Limited uptake of HTA in policy decisions and low stakeholder engagement*

Respondents highlighted policy inertia and low stakeholder involvement as significant weaknesses, with 68% rating the issue “relevant” or “very relevant.” Stagnation remained high (45%), with only 32% noting improvement and 23% perceiving worsening. Comments detailed minimal use of HTA outputs in actual decision-making processes and limited involvement of diverse stakeholders such as patients, clinicians, and payers.

*W8. Low public and patient awareness, and reluctance to engage in shared decision-making*

Awareness and engagement were judged “relevant” or “very relevant” by nearly 68%, with 27% reporting “somehow relevant” and only 5% “not at all.” Change was again limited: half reported no shift, with 27% improvement and 23% worsening. Qualitative feedback described public and patient involvement in HTA and health policy as largely absent, though exceptions exist for rare diseases. Respondents repeatedly cited the lack of formal mechanisms for shared decision-making.

#### Opportunities (Table S3)

*O1. Growing national and professional interest in evidence-based medicine*

This was strongly supported, with 86% of respondents rating this opportunity as “relevant” or “very relevant.” The majority (77%) also perceived improvement in recent years. Comments reflect increased attention to EBM, especially in academic settings and clinical practice, but note that institutionalization of EBM within national HTA frameworks is still evolving.

*O2. Rising healthcare costs, prompting demand for resource optimization*

There was marked consensus on the relevance of this pressure: 91% deemed rising healthcare costs “relevant” or “very relevant” as a driver for HTA adoption. Most respondents (45%) reported improved opportunities, while others saw either stagnation or increased challenges. Comments from Türkiye highlight how increased financial pressures in the health system are making HTA a critical tool for sustaining service delivery.

*O3. WHO and World Bank support for integrating HTA into Universal Health Coverage strategies*

Most participants (59%) rated this statement as “relevant” or “very relevant.” Perceptions of change were more mixed: 41% said opportunities had improved, while 41% saw no change and 18% reported worsening. Comments observed that while support from international agencies exists, the integration of HTA into major health coverage strategies is not yet fully realized and stakeholder engagement remains low.

*O4. Potential for joint policy advocacy and regional position statements on HTA*

Most respondents (73%) saw advocacy and position statements as “relevant” or “very relevant,” but 68% reported stability with little improvement. Several respondents highlighted Türkiye’s potential as a regional leader but noted this has yet to be effectively harnessed for joint advocacy or policymaking.

*O5. Educational exchange opportunities and training via webinars, workshops, etc.*

A high proportion (82%) considered educational exchanges “relevant” or “very relevant,” with 59% indicating improved opportunity. Comments emphasized that while webinars and workshops are widely available, targeted institutional uptake and better organization are needed to maximize capacity-building and impact.

*O6. Existing trained personnel and academic infrastructure in some areas*

Support for leveraged capacity and infrastructure was moderate, with 64% finding this “relevant” or “very relevant.” Change perceptions were split; half noted little progress, while 45% indicated improvement. Respondents explained that in countries like Türkiye, HTA expertise is mostly found in individuals with international training, as local academic infrastructure for HTA remains limited.

*O7. International collaboration for sharing experience and technical support*

Most respondents (77%) judged international collaboration to be a “relevant” or “very relevant” opportunity. Positive change was reported by half, while the remainder saw little movement. Comments pointed to project-based and ad-hoc collaborations (especially at the EU level) as examples but stressed the need for more systematic and sustained multinational partnerships.

*O8. Increasing demand from patient groups for evidence in healthcare decisions*

Most saw rising patient demand for evidence-based care as an opportunity (68% “relevant” or “very relevant”). 59% noted improvement, but a third saw no change. Comments described minimal organized patient demand in contexts like Türkiye and highlighted persistent gaps in advocacy, organization, and awareness among patient groups.

#### Threats (Table S4)

*T1. Insufficient funding for EBM/HTA capacity development*

This threat was strongly endorsed, with 82% of respondents considering insufficient funding “relevant” or “very relevant.” Most (50%) reported stagnation, 32% noted worsening, and only 18% saw improvement. Comments highlighted the chronic reliance on short-term or externally funded projects rather than sustainable national financing for HTA/EBM capacity.

*T2. Low awareness or resistance among policy-makers and clinicians*

Most respondents (77%) viewed this threat as “relevant” or “very relevant,” with half reporting unchanged conditions and a minority either worsening or improving. Comments noted persistent low awareness among policy-makers and minimal integration into daily clinical practice, describing interaction levels between HTA experts and decision-makers as suboptimal.

*T3. Strong influence of the pharmaceutical and medical device industries*

Majority agreement (82%) placed industry influence as a principal threat. Most observed little change, but 32% saw improvement and 18% noted further worsening. Comments highlighted gaps in HTA structures and oversight, allowing non-systematic evidence to be outweighed by commercial interests in market access and reimbursement decision-making.

*T4. Lack of incentives for physicians to adopt EBM practices*

Respondents broadly recognized lack of incentives as a threat (77%), with half reporting stagnation, 32% improvement, and 14% worsening. Comments indicated that most physicians in Türkiye apply evidence informally and that there are no requirements or formal incentives in place for regular EBM adoption.

*T5. Cultural resistance from senior professionals opposing EBM-driven change*

A relative majority (68%) judged cultural resistance as “relevant” or “somehow relevant,” with most reporting stasis. Qualitative data describe reliance on traditional hierarchies and outdated practices among senior staff, limiting adoption of contemporary EBM/HTA approaches and slowing systemic change.

*T6. Weak professional societies in the country limiting advocacy potential*

Most respondents saw weakness in professional societies as a threat (59%), with 32% “somehow relevant.” Only 14% found this “not at all” relevant. Two-thirds indicated no change in advocacy potential, while others saw minor improvement or worsening. Observers noted some EBM-focused associations exist, but their role in HTA advocacy is limited and underdeveloped.

*T7. Discontinuity of personnel and projects, risking sustainability*

This threat was endorsed by 64% as “very relevant” or “relevant,” with 32% “somehow relevant.” Most reported no progress in stability or continuity, and roughly a third noted worsening sustainability. Comments described how HTA development in Türkiye and other countries often relies on motivated individuals, with frequent discontinuities when staff changes or projects end.

*T8. Mismatch between national ambitions and existing capacities*

Majority agreement (64%) placed the capacity gap as a key threat, with one-third “somehow relevant.” While half reported stasis, 32% saw worsening and fewer than 10% noted improvement. Participants commented on ambitious aspirations for regional HTA leadership, juxtaposed with limited current institutional and human capacity.

**Table S1:** Elements of strength.

|  | *Not at all* | *Somehow relevant* | *Relevant* | *Very Relevant* |
| --- | --- | --- | --- | --- |
| S1. Strong interest in regional collaboration, including cross-country HTA activities | 4,5% | 22,7% | 31,8% | **40,9%** |
| S2. Political and institutional will through WHA resolution promoting HTA | 22,7% | 18,2% | **50%** | 9,1% |
| S3. Availability of online resources and educational materials | 0% | 27,3% | **50%** | 22,7% |
| S4. Existing training programs (e.g. pharmacoinformatics, critical appraisal) for health professionals in some countries | 9,1% | 22,7% | **50%** | 18,2% |
| S5. Capacity-building efforts supported by both national and regional actors | 9,1% | 27,3% | **31,8%** | **31,8%** |
| S6. Presence of Evidence-Based Medicine (EBM) associations in the country offering peer motivation | 18,2% | 13,6% | 31,8% | **36,4%** |
| S7. Global support andartnerships (e.g. World Health Organization, World Bank) backing HTA expansion | 13,6% | 13,6% | **45,5%** | 27,3% |

**Table S2:** Elements of weakness.

|  | *Not at all* | *Somehow relevant* | *Relevant* | *Very Relevant* |
| --- | --- | --- | --- | --- |
| W1. Shortage of trained professionals in Evidence-Based Medicine and HTA | 9,1% | 9,1% | **45,5%** | 36,4% |
| W2. Lack of standardized, certified HTA/Evidence-based Medicine programs across country | 13,6% | 13,6% | **40,9%** | 31,8% |
| W3. Absence of Evidence-Based Medicine in academic curricula at all educational levels | 18,2% | 22,7% | **36,4%** | 22,7% |
| W4. Over-reliance on external expertise due to lack of national capacity | **36,4%** | 13,6% | 27,3% | 22,7% |
| W5. Language barriers limiting accessibility to core documents and guidelines | 26,3% | 9,1% | **36,4%** | 27,3% |
| W6. Missing or weak legal frameworks for institutionalizing HTA | 18,2% | 18,2% | **40,9%** | 22,7% |
| W7. Limited uptake of HTA in policy decisions and low stakeholder engagement | 22,7% | 9,1% | **40,9%** | 27,3% |
| W8. Low public and patient awareness, and reluctance to engage in shared decision- making | 4,5% | 27,3% | 27,3% | **40,9%** |

**Table S3:** Elements of opportunity.

|  | *Not at all* | *Somehow relevant* | *Relevant* | *Very Relevant* |
| --- | --- | --- | --- | --- |
| O1. Growing national and professional interest in evidence-based medicine | 4,5% | 9,1% | **63,6%** | 22,7% |
| O2. Rising healthcare costs, prompting demand for resource optimization | 4,5% | 4,5% | 36,4% | **54,5%** |
| O3. World Health Organization and World Bank support for integrating HTA into Universal Health Coverage strategies | 9,1% | 31,8% | **40,9%** | 18,2% |
| O4. Potential for joint policy advocacy and regional position statements on HTA | 0% | 27,3% | **59,1%** | 13,6% |
| O5. Educational exchange opportunities and training via webinars, workshops, etc. | 4,5% | 13,6% | **40,9%** | **40,9%** |
| O6. Existing trained personnel and academic infrastructure in some areas | 9,1% | 27,3% | **40,9%** | 22,7% |
| O7. International collaboration for sharing experience and technical support | 9,1% | 13,6% | **50%** | 27,3% |
| O8. Increasing demand from patient groups for evidence in healthcare decisions | 4,5% | 27,3% | **40,9%** | 27,3% |

**Table S4:** Elements of threat.

|  | *Not at all* | *Somehow relevant* | *Relevant* | *Very Relevant* |
| --- | --- | --- | --- | --- |
| T1. Insufficient funding for Evidence-Based Medicine/HTA capacity development | 0% | 18,2% | 27,3% | **54,5%** |
| T2. Low awareness or resistance among policy-makers and clinicians | 4,5% | 18,2% | **40,9%** | 36,4% |
| T3. Strong influence of the pharmaceutical and medical device industries | 4,5% | 13,6% | **59,1%** | 22,7% |
| T4. Lack of incentives for physicians to adopt Evidence-Based Medicine practices | 4,5% | 18,2% | **45,5%** | 31,8% |
| T5. Cultural resistance from senior professionals opposing Evidence-Based Medicine- driven change | 4,5% | **50,0%** | 27,3% | 18,2% |
| T6. Weak professional societies in the country limiting advocacy potential | 13,6% | 27,3% | **45,5%** | 13,6% |
| T7. Discontinuity of personnel and projects, risking sustainability | 4,5% | 31,8% | 22,7% | **40,9%** |
| T8. Mismatch between national ambitions and existing capacities | 4,5% | 31,8% | 27,3% | **36,4%** |
